# Is Screening or Resection of “Minimal” (Early) Hepatocellular Carcinoma Justified?

**DOI:** 10.1155/1990/10792

**Published:** 1990

**Authors:** B. Lanois

**Affiliations:** Centre Hospitalier & Universitaire de Rennes Rue Henri le Guilloux Rennes Cedex 35033 France


*HPB Surgery*, 1990, Vol. 3, pp. 73–78
Reprints available directly from the publisher
Photocopying permitted by license only

© 1990 Harwood Academic Publishers GmbH
Printed in the United Kingdom

### HPB INTERNATIONAL

**EDITORIAL & ABSTRACTING SERVICE**

JOHN TERBLANCHE, EDITOR
Department of Surgery,
Medical School · Observatory 7925
Cape Town · South Africa
Telephone: (021) 47-1250 Ext. 229
Telefax (021) 47-8955

### IS SCREENING OR RESECTION OF "MINIMAL" (EARLY) HEPATOCELLULAR CARCINOMA JUSTIFIED?

### ABSTRACT

*Cottone M, Virdone R, Fusco G, Orlando A, Turri M, Caltagirone M, Maringhini A, Sciarrino E, Demma I, Nicoli N, Tine F, Sammarco S, and Pagliaro L. Asymptomatic Hepatocellular Carcinoma in Child's A Cirrhosis: A comparison of natural history and surgical treatment. Gastroenterology 1989;96: 1566–1571.*

**The present study deals with the natural history of 37 asymptomatic patients with cirrhosis and hepatocellular carcinoma, 25 with 2–9-cm tumours who were not surgically treated (first group) and 12 with tumours smaller than 4 cm who underwent resection (second group). All patients were in Child's A class. Two-year survival (according to life-table analysis by the Kaplan-Meier method) was 50% in the first group and 39% in the second group. This difference was not significant. In the first group no relation was found between survival and initial tumour size or $\alpha$-fetoprotein levels. Ultrasound examinations at 3-month intervals revealed the following patterns of tumour growth: (a) no significant growth during the follow-up (9 patients); (b) significant growth (tumour size at least doubling) only in the final stage of the disease (11 patients); (c) initial significant growth followed by a period of no increase in size (5 patients). These findings show that in our geographical area (a) 2-yr survival of untreated asymptomatic patients with hepatocellular carcinoma associated with cirrhosis does not differ from that of similar patients undergoing resection and (b) the tumour can exhibit long periods of no growth alternating with periods of exponential growth.**

Reprinted with permission from the authors of the above article from the journal Gastroenterology. Copyright 1989 by The American Gastroenterological Association.

### PAPER DISCUSSION

KEYWORDS: Hepatocellular carcinoma, screening, liver resection

The work of Cottone *et al.* entitled : "Asymptomatic hepatocellular carcinoma in Child's A cirrhosis : a comparison of natural history and surgical treatment" is of interest in that it reviews the natural history of hepatocellular carcinoma (HCC), with special emphasis on asymptomatic HCC, and compares survival for untreated and operated patients. Studies to date have mainly been of Asian origin and have confirmed the value of surgical treatment for HCC with cirrhosis. As a consequence, systematic screening was introduced, resulting in early detection of such tumours.

In Shangaï, 1,967,511 persons were screened by AFP tests between 1971 and 1976; of the 300 cases picked up, 44.7% were asymptomatic with a 3-year survival of 57.1% after resection[1]. In the Quidong region, 1,223,912 people were screened during the period 1974–1979 and 475 cases of NCC were detected: 35.2% of these were asymptomatic and 2-year survival for resected patients was 6.9%[1].

Systematic screening seemed justified particularly because non-surgical treatment gave mediocre results: a 2-year survival of 6% for a population of 502 patients in Okuda's series[1]. In our own series, survival was 22% and 5% at 1 and 2 years respectively for patients who had had palliation and no excision[2].

Few studies, however, have examined the natural history of HCC. In 1986, Ebara[3] published a natural history of minute hepatocellular carcinoma (smaller than 3 centimetres), with cirrhosis. Survival at 1, 2 and 3 years was 75.6%, 48.1% and 12.8% respectively. Survival for Mario Cottone's untreated patients is very close to these figures, but he does not give the results at 3 years. Moreover, the comparison between operated and non-operated patients only covers a period of 2 years. Nagasue [4] compared his results after resection with Ebar's work on natural history and showed that the two curves were parallel up to 2 years. Survival at 3 years, on the other hand, is 58.8% after resection as against 12.8%.

Future studies on natural history must be pursued for more than 3 years, and there should also be precise histological studies of the cirrhosis and the liver tumour. The diagnosis of cirrhosis is based on 3 criteria: annular fibrosis, parenchymatous nodules and disorganized vascular architecture. The authors insist that death is due to complications caused by cirrhosis (variceal haemorrhage, hepatic failure), but histological proof is needed.

In our series of 85 HCC with diseased liver [2], there were 18 cases of liver fibrosis and 2 of regenerative nodular hyperplasia. The liver was normal in 2 cases, despite a positive HBS antigen. The diagnosis of cirrhosis can only be established at distance from the malignant lesion. Prognosis may be favourable if the diagnosis of cirrhosis is erroneous and the patient has a different liver disease.

The diagnosis of HCC is itself difficult: abnormal ultrasound or CT scans may turn out to correspond to hyperplastic nodules or non-malignant lesions[1–2]. Liver needle biopsy is inadequate for differentiation (2 cases out of 5 in our series). In cases of excision, it can be very difficult, if not impossible, to make a diagnosis between a hyperplastic nodule and a welldifferentiated HCC. Four patients in our series had hepatic resection for cirrhotic HCC with positive needle biopsy but this was not confirmed at definitive histological examination. Okuda[1] made the same observation the same year. As he remarked, with such experiences accumulating at various medical centers, a serious question has been raised as to how to differentiate HCC from such benign lesions and whether or not the seemingly benign lesions were preneoplastic. Arakawa described 17 small lesions detected by imaging diagnosis and resected from 10 patients with cirrhosis. Four were considered to be similar to adenomatous hyperplasia as described by Edmonson and eight were equivocal, either an adenomatous hyperplastic nodule undergoing malignant transformation or extremely well differentiated malignant lesions[1]. It is therefore easy to understand how difficult it is to study the natural history of HCC with cirrhosis without histological confirmation by excision or extended biopsy

It is also well-established, however, that a capsule constitutes a favourable prognosis factor. The existence of a capsule has been considered characteristic of Asian tumors. In fact, it exists in 30% to 50% of HCCs seen in Europe[2–5]. Gozetti [5] in particular has insisted on the fact that patients who lived beyond 12 months and who presented no ultrasound evidence of recurrence had a well-defined peritumoral capsule observable both microscopically at ultrasonography and at histological examination.

The authors have not included this precision. It has been suggested that an integral capsule is indicative of a slow-growing tumor, therefore the lesion remains limited up to the moment that the tumor projects outside of its capsule. Finally, the authors observed two diffuse forms in the end follow-up for untreated HCC. It is surprising that they did not find multiple lesions. Lipiodol arteriography should at least have been performed during the first assessment of these lesions[1].

It is nevertheless true that these asymptomatic tumours probably have a more favourable prognosis. Before ultrasonography and CT scanning came into use, HCC was discovered incidentally on operative specimens from total hepatectomy with liver transplantation. Long-term survival is significantly longer than for other liver transplantations for cancer.

Professor B. Lanois
Centre Hospitalier & Universitaire de Rennes
Rue Henri le Guilloux
35033 Rennes Cedex
France

### REFERENCES

1. Okuda, K. (1986) Early recognition of hepatocellular carcinoma. *Hepatology,* **6**, 729–738.
2. Launois, B., Bourdonnec, P., N'Guyen, T., Deugnier, Y., Campion, J.P., de Chateaubriant, P. (1986) Le traitement chirurgical des hépatomes sur cirrhose. *Nouv. Presse Med.* **15**, 2235–2238.
3. Ebara, M., Ohto, M., Shinaga, W.A.T. *et al.* (1986) Natural history of minute hepatocellular carcinoma smaller than three centimetres complicating cirrhosis: A study in 22 patients. *Gastroenterology.* **90**, 289–298.
4. Nagasue, N., Yukaya, M., Chang, Y.C., Ogawa, Y., Ota, N., Kimura, M. and Makamura, T. (1987) Appraisal of hepatic resection in the treatment of minute hepatocellular carcinoma associated with liver cirrhosis. *Br. J. Surg.,* **74**, 836–838.
5. Gozzetti, G., Mazzzotti, A., Cavallari, A., Bellusci, R., Bolondi, L., Grigioni, W., Bragaglia, R., Grazi, G.L. and de Raffele, E. (1988) Clinical experience with hepatic resection for hepatocellular carcinoma in patients with cirrhosis. *Surg. Gynecol. Obstet.,* **166**, 503–510.

